# Metaproteomic analysis of Chesapeake Bay microbial communities

**DOI:** 10.1186/1746-1448-1-7

**Published:** 2005-08-19

**Authors:** Jinjun Kan, Thomas E Hanson, Joy M Ginter, Kui Wang, Feng Chen

**Affiliations:** 1 Center of Marine Biotechnology, University of Maryland Biotechnology Institute, Baltimore, MD 21202, USA; 2 Graduate College of Marine Studies and Delaware Biotechnology Institute, University of Delaware, Newark, DE 19711, USA; 3 Department of Chemistry and Biochemistry, University of Delaware, Newark, DE 19716, USA

## Abstract

**Background:**

Natural microbial communities are extremely complex and dynamic systems in terms of their population structure and functions. However, little is known about the *in situ *functions of the microbial communities.

**Results:**

This study describes the application of proteomic approaches (metaproteomics) to observe expressed protein profiles of natural microbial communities (metaproteomes). The technique was validated using a constructed community and subsequently used to analyze Chesapeake Bay microbial community (0.2 to 3.0 μm) metaproteomes. Chesapeake Bay metaproteomes contained proteins from pI 4–8 with apparent molecular masses between 10–80 kDa. Replicated middle Bay metaproteomes shared ~92% of all detected spots, but only shared 30% and 70% of common protein spots with upper and lower Bay metaproteomes. MALDI-TOF analysis of highly expressed proteins produced no significant matches to known proteins. Three Chesapeake Bay proteins were tentatively identified by LC-MS/MS sequencing coupled with MS-BLAST searching. The proteins identified were of marine microbial origin and correlated with abundant Chesapeake Bay microbial lineages, *Bacteroides *and α-proteobacteria.

**Conclusion:**

Our results represent the first metaproteomic study of aquatic microbial assemblages and demonstrate the potential of metaproteomic approaches to link metagenomic data, taxonomic diversity, functional diversity and biological processes in natural environments.

## Background

Bacterioplankton contribute significantly to both primary production and biomass in the ocean and coastal water [1,2]. With an average concentration of approximately 10^6 ^cells ml^-1^, bacterioplankton is an important catalyst of biogeochemical processes including oceanic carbon and nitrogen cycles [3,4]. Studying bacterioplankton is challenging because most groups either have never been cultivated [5,6] or grow to very low density in the laboratory [7]. Culture-independent molecular approaches have indicated that environmental bacterial communities are more complex and diverse than previously thought [5,6,8]. Metagenomics is the direct cloning, sequencing, assembly and annotation of DNA from microbial communities and has been applied to waters, soils and extreme environments [9-12]. A recent metagenomic study of the Sargasso Sea revealed that substantial complex microbial diversity exists in the ocean: 148 novel bacterial phylotypes and more than a million of previously unknown genes were discovered and annotated [12].

As genomic data accumulates from pure cultures and environmental communities, it becomes critical to understand gene expression and protein function. While metagenome sequences provide valuable information on potential functions, accurately predicting ecological function from sequence is nearly impossible without information on what proteins are synthesized under specific conditions [13-15]. To address this question, post-genomic molecular approaches such as microarrays to monitor mRNA abundance [16] have been developed. In addition, as proteins/proteomes are the ultimate functional products of genes/genomes, proteomic studies of microbial communities (metaproteomics) are an obvious approach to advance our understanding of microbial community function.

Metaproteomics can provide a direct measurement of functional gene expression in terms of the presence, relative abundance and modification state of proteins [17,18]. Proteomics and metaproteomics rely on two-dimensional gel electrophoresis (2D-PAGE) coupled with mass spectrometry (MS) based protein identification relying on mass based (MALDI-TOF MS) or sequence based (LC-ESI-MS/MS) methods. These techniques have only been applied in limited scope to environmental microbial communities. One-dimensional gel electrophoresis (1D-PAGE) coupled with radioactive labelling or enzymatic activity assay has been used to study proteins induced in response to environmental stresses [19,20]. However, little concrete information on the sequences or identities of induced proteins emerged from these studies. A metaproteomic approach was applied to a laboratory-scale activated sludge bioreactor resulting in the identification of three highly expressed proteins presumably originating from an uncultured *Rhodocyclus-*type polyphosphate – accumulating organism [18]. More recently, using genomic and mass spectrometry-based proteomic methods, metaproteomes from an acid mine drainage (AMD) microbial biofilm community have been identified and linked their *in situ *functions to the challenging environments [21]. However, all these studies are dealing with low-complexity microbial communities. So far, no studies have yet applied proteomic approaches to natural aquatic microbial communities.

**Figure 1:**
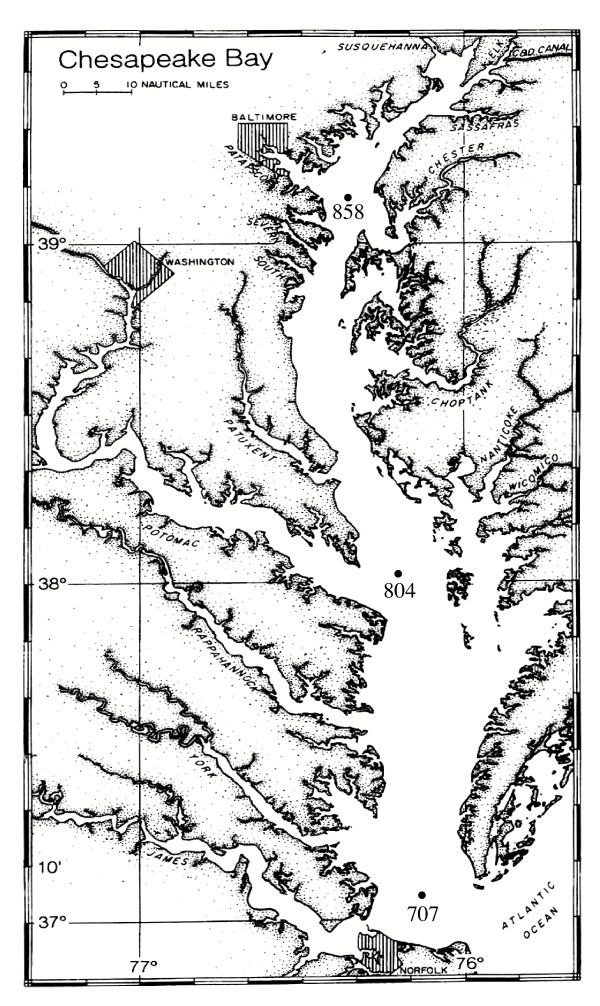
Metaproteome sampling stations at the Chesapeake Bay.

Estuaries represent one of the most complex and productive ecosystems. The Chesapeake Bay is the largest estuary in United States (Fig. 1). It has received a great deal of attention because of its large geographic span and economic significance. With strong environmental gradients, it provides an ideal model system for integrated investigations on composition and function of microbial communities. In this study, we developed a metaproteomic approach to document microbial community protein profiles along a transect of the Chesapeake Bay. Significant differences were noted between proteomes collected at different sites and metaproteome patterns accurately predicted the relationship of sites as determined by 16S rRNA gene PCR-DGGE (denaturing gradient gel electrophoresis). Furthermore, proteins identified from Chesapeake Bay samples appeared to originate from marine bacterioplankton. This study demonstrates that metaproteomic approaches can be successfully applied to naturally occurring and complex microbial communities in their native habitats.

## Results

### Microbial community collection

Epifluorescence microscopic counts showed that concentrated microbial communities mainly contained free-living bacteria (~ 95%). The recovery efficiency of bacterial cells using the tangential flow ultrafiltration system was 75 ± 5% (data not shown). With the average concentration of 2.5 × 10^6 ^cells ml^-1 ^in the starting water samples, the density of microbial cells in the ultrafiltration retentate was about 2.5 × 10^8 ^cells ml^-1^. Thus, about 3.75 × 10^10 ^cells were analyzed in each sample. Extracts typically contained between 140 and 192 μg of protein giving a value range of 3.7 × 10^-15 ^to 5.1 × 10^-15 ^g protein cell^-1^. This value is significantly lower than that determined for cultured strains in this study and in general for marine bacteria (60–330 × 10^-15 ^g protein cell^-1^, [22]). It remains to be determined whether this discrepancy indicates that the extraction protocol needs further optimization or is a fundamental property of microbial cells in environmental samples.

### 1D-PAGE analysis of proteins from isolated bacterial strains and environmental samples

**Figure 2:**
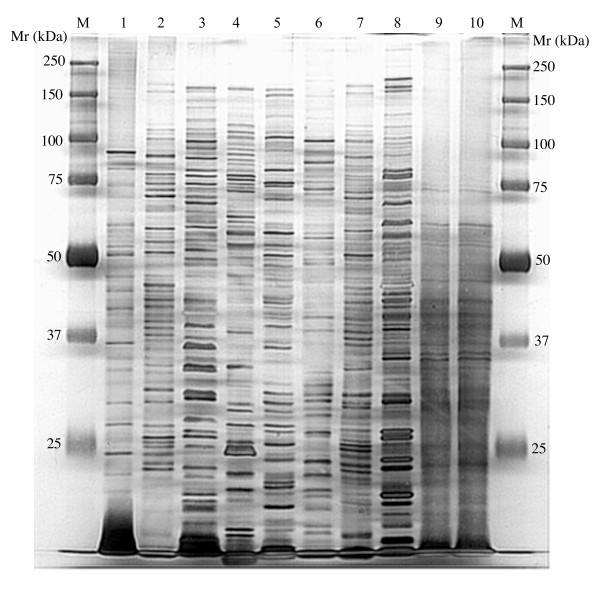
**1D-PAGE patterns of total proteins obtained from 8 different bacteria isolated from Baltimore Inner Harbor**. M, Marker; Mr, molecular weight; Lanes 1 – 8 correspond to *Vibrio vulnificus*, Marine *Bacillus *sp., *Marinomonas *sp., *Psychrobacter pacificens, Pseudomonas *sp., *Pseudoalteromonas *sp., *Shewanella *sp., and *Hahella *sp.. Lanes 9 and 10 are duplicated environmental microbial communities. For each lane, 20 μg of protein is loaded and the gel is stained by silver staining.

Individual proteins from cultivated marine bacteria were well resolved by 1D-PAGE and produced distinct patterns when 8 Chesapeake Bay bacterial isolates were compared (Fig. 2). The observed molecular masses ranged from ~10 to 250 kDa (Fig. 2, lanes 1–8) whereas proteins from microbial community samples were < 80 kDa (Fig. 2, lanes 9 and 10). Overall resolution was much poorer in community samples as evidenced by less sharply defined bands in these samples. This blurring effect was also noted in a very simple mixed microbial community described below and was not dependent on sampling manipulations (data not shown).

### Analysis of isolated strains and artificial mixed communities

Artificial community consisting of *Chlorobium tepidum *strain WT2321, *Escherichia coli *strain JM109 and an uncharacterized strain of *Pseudomonas fluorescens *was analyzed by 2D-PAGE. Preliminary experiments indicated that a 300 ml sample containing 1 × 10^7 ^cells per ml of the community could be successfully analyzed by 2D-PAGE. Analysis by 1D-PAGE afforded greater sensitivity, ~1 × 10^4 ^cells per ml, but resolution of individual bands was poor as noted above. Protein assays on samples of the community before dilution and recovery and after indicated that the metaproteomic sample preparation recovered ~ 30% of the total microbial protein present in the original community sample.

**Figure 3:**
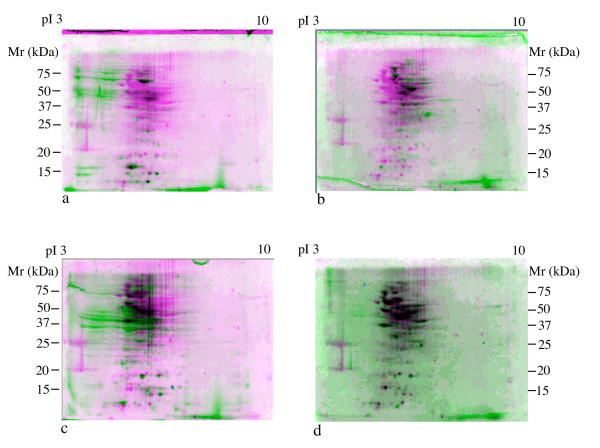
**The harvesting protocol for microbial communities does not bias against different types of bacteria**. Proteomes of *Chlorobium tepidum *(a), *Escherichia coli *(b) and *Pseudomonas fluorescens *(c) and the metaproteomes of an artificially constructed community containing all three organisms (d) were overlain and compared to the metaproteomes of the artificial community after dilution and recovery using Compugen Z3 software. Green or pink colored protein spots are unmatched. Gray or black spots are matched. Total 100 μg proteins are loaded on each polyacrylamide gel and the gels are stained by SYPRO Ruby. pI, isoelectric point; Mr, molecular weight.

Typical results from a 2D-PAGE experiment are shown in Fig. 3. The overlays indicate that 2D-PAGE patterns from single strains of community members only match a fraction of protein spots present in the mock metaproteome sample (Fig. 3a–d). This is qualitatively observed as a large number of green or pink protein spots in the overlay views showing unmatched protein spots. Each individual strain is expected to contribute only one third of the protein content of the community. In contrast, when a sample of the community prior to dilution and recovery is compared to a mock metaproteome that had been subjected to sample handling protocols, almost perfect matching of the samples is seen as evidenced by the large proportion of dark grey to black spots (Fig. 3d) when these images are overlain. Thus, no individual member of the community, which covers the range of cell sizes in the environmental samples, is selectively excluded by the sampling protocol.

### Extraction of metaproteomes from the Chesapeake Bay

In this study, in order to optimize the protein extraction of aquatic microbial communities, different protocols that varied all steps in protein extraction and purification were tested including (i) sample collection (filtration on membrane filter, tangential flow concentration with centrifugation); (ii) washing buffer to remove ambient salts and polysaccharides; (iii) extraction buffer (standard lysis buffer, SDS-PAGE buffer, urea-thiourea-CHAPS buffer); (iv) reducing agent (dithiothreitol (DTT) vs. tributyl phosphine (TBP));(v) cell lysis method (freeze-thaw, French pressure cell); (vi) protein precipitation (acetone vs. TCA); (vii) IPG strip range (pH 3–10 vs. pH 4–7); and (viii) staining method (Commassie blue, silver, SYPRO Ruby). From these trials, the following protocol emerged: (i) tangential flow concentration with centrifugation; (ii) TS washing buffer (Tris 10 mM, Sucrose 250 mM); (iii) urea-thiourea-CHAPS lysis buffer with TBP; (iv) lysis via French pressure cell; (v) TCA precipitation; (vi) First dimension pH 4–7 IPG strip; (vii) SYPRO Ruby staining. However, given the indigenous characteristics among diverse microbial communities, extraction of metaproteomes may vary by site, time and experiment as well.

### Quantitative Comparison of Chesapeake Bay Metaproteome Samples

**Figure 4:**
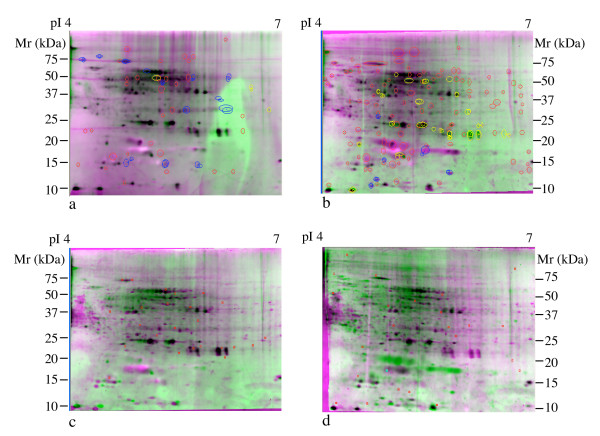
**Comparisons of Chesapeake Bay metaproteomes**. (a) Independent samples from Station 804, 804a and 804b; (b) Station 804a vs. Station 707; (c) Station 804a vs. Station 858; (d) Station 707 vs. 858. Image overlays were constructed with Compugen Z3 software. Spots circled in red are unmatched, those in yellow and blue are differentially expressed at a level of ≥ 3-fold between images. No unmatched or differential spots are shown in c and d because software based matching of these images failed. Red marks in panels c and d are alignment points used to produce the pictured overlay. Quantitative results of matching are reported in Table 1. Total 100 μg proteins are loaded on each polyacrylamide gel and the gels are stained by SYPRO Ruby. pI, isoelectric point; Mr, molecular weight.

Metaproteome images from different Chesapeake Bay stations in the upper (station 858), middle (station 804, replicates a and b) and lower Bay (station 707) were compared (Fig. 4a–d). A number of protein spots were shared by all samples. Some of these are proteins present in RNase, Dnase and protease inhibitor cocktail in the extraction buffer (data not shown), but a number of proteins appear to be common in all samples examined. These are black to dark grey spots in the image overlays (Fig. 4a–d). A first level of quantitative comparison determined the specific numbers of protein spots shared between samples (Table 1). The total number of spots compared for each sample is relatively low as the analysis was restricted to spots with sufficient quality and intensify to permit subsequent attempts at protein identification. As expected, replicate metaproteome images from the middle Bay are more similar to one another than the metaproteomes of other stations, sharing ~92 % of all detected spots. Furthermore, the lower and middle Bay metaproteomes are significantly more similar to one another than either is to the upper Bay metaproteomes with ~70 % of all detected spots in common. The upper Bay metaproteomes only shared about ~30 % of detected spots with either the middle or lower Bay metaproteomes.

**Table 1:** Quantitative comparison of Chesapeake Bay metaproteomes.

Samples compared	spots^a^	unmatched^a^	differential^a,b^
804a	207	7	3
vs. 804b	189	26	13
	396	33 (8.3 %)	16(4.0%)
			
804a	207	37	23
vs. 707	198	86	6
	405	123 (30.3 %)	29(7.1 %)
			
804a	207	156^C^	--^d^
vs. 858	155	104^c^	--
	362	160 (71.8 %)	--
			
707	198	142^b^	--
vs. 858	155	99^b^	--
	353	241 (68.3 %)	--

^a^-Spots from first gel, second gel and the sum are listed. Numbers in parentheses show the percentage of the total.

^b^-Matched spots that are ≥ 3-fold more intense than the comparative image.

^c^-Estimated by manual comparison of detected spots. Software was unable to match images.

^d^-No differential comparison possible as software based matching failed.

Relative spot intensity was extracted from comparisons of middle Bay to middle Bay and middle Bay with lower Bay metaproteome images. This was not possible with the upper Bay sample as manual matching was employed due to the low level of similarity between samples. Again, as expected, the number of differentially expressed proteins (≥ 3-fold change in matched spot intensity) was nearly twice as large when comparing middle Bay to a lower Bay metaproteomes as when comparing the replicated middle Bay samples (Table 1). These results indicate that both qualitative and highly quantitative comparisons between sites and between time series samples at the same site will be possible using the approaches developed in this study.

### Identification of Proteins in Chesapeake Bay Metaproteomes

A total of 41 protein spots were excised from a number of 2-D gels reflecting various molecular weights, charges and relative abundance. Following MALDI-TOF MS, seven spots failed to yield interpretable MS profiles, while the remaining 34 proteins exhibited clear and distinct MS peaks. Database searches using the MASCOT search engine with varying parameter settings (peptide mass tolerance from 0.5 to 3 Da, missed cleavages from 1 up to 5) produced no significant matches for these 34 proteins. Subsequent publications from other laboratories and our own simulations using known protein sequences [[23,24], Hanson, unpublished data] suggest that greater than 97 % amino acid sequence identity is required to provide a positive match when searching with MALDI-TOF MS data.

**Figure 5:**
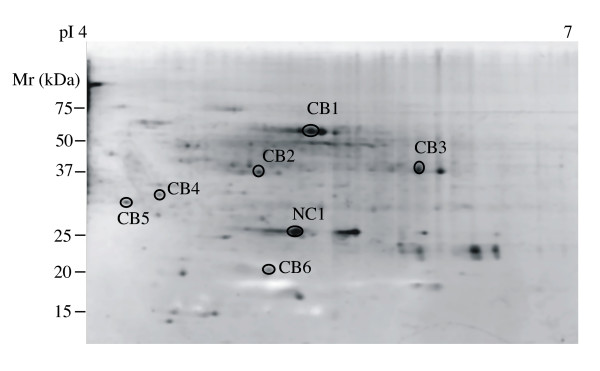
**Proteins selected for identification from middle Chesapeake Bay (station 804)**. Total 100 μg protein are loaded on polyacrylamide gel and the gel is stained by SYPRO Ruby. CBl-CB6 samples are common to Chesapeake Bay stations while NC1 is found on negative control gels containing DNase, RNase and protease inhibitors. Results of protein identification are reported in Table 2 and 3. pI, isoelectric point; Mr, molecular weight.

Seven individual proteins (Fig. 5) isolated from middle Chesapeake Bay (station 804) metaproteome samples were further analyzed by both MALDI-TOF MS and LC-MS/MS sequencing coupled to MS-BLAST searching (Table 2). MALDI-TOF MS failed to provide identification for any of these samples, similar to the samples described above. LC-MS/MS based searches provided tentative identities for three Chesapeake Bay metaproteome samples. These were identified as homologues of hypothetical proteins annotated in the recently reported Sargasso Sea metagenome [12]. Information on potential functions of these proteins was obtained by downloading the full length proteins from the Sargasso Sea database and searching them against known databases by BLASTP (Table 3). The Sargasso Sea metagenome hypothetical protein corresponding to sample CB1 is not significantly similar to any known proteins in sequence databases. Sample CB3 may correspond to subunit 7 of the NADH:ubiquinone oxidoreductase (complex I) while sample CB6 is similar to a family of predicted aminopeptidases with unspecified functional significance. The tandem mass spectra of samples CB2, CB3 and CB5 had no match with any known proteins or hit keratin and bovine serum albumin that possibly came from background.

**Table 2:** Identification of proteins from Chesapeake Bay station 804 metaproteomes (Fig. 5).

Sample	pI	MW	MALDIID?^a^	MS/MS ID?^b^	Peptides Matched	Score^c^	Accession
NC1	5.1	29 kDa	No	No	-	-	-
CB1	5.3	60 kDa	No	Sargasso sea metagenome	2	110	EAH98995.1
CB2	4.9	40 kDa	No	Bovine serum albumin	2	138	P02769
CB3	5.7	42 kDa	No	Sargasso sea metagenome	3	116	EAH45127.1
CB4	4.4	35 kDa	No	Keratin	2	117	Q9DCV7
CB5	4.2	33 kDa	No	No -	-	-	-
CB6	5.0	20 kDa	No	Sargasso sea metagenome	2	88	EAC65279.1

^a^-MASCOT search as described in Materials and Methods.

^b^-MS-BLAST search as described in Materials and Methods.

^c^-For a description of scoring, see reference 27.

**Table 3:** BLASTP analysis of Sargasso sea metagenome hits.

Sample	Accession	Best hit	E-value	Organism	Accession
CB1	EAH98995.1	Hypothetical protein	0.47	*Plasmodium berghei*	CAI00437
CB3	EAH45127.1	NADH:UQ oxidoreductase (49 kDa, subunit 7)	1 × 10^-63^	*Cytophaga hutchinsonii*	ZP_00309190
CB6	EAC65279.1	Predicted aminopeptidase	2 × 10^-16^	*Novosphingobium aromaticivorans*	ZP_00305215

## Discussion

In this study, we deliberately focused on exploring the proteome profiles from bacterioplankton communities between 0.2 and 3.0 microns in size by the choice of prefiltration and ultrafiltration cut-off sizes. Although the epifluorescence microscopy observation confirmed that the major components are bacterioplankton (~95%), small numbers of eukaryotic microbes were possibly included. These likely did not affect the overall protein profiles observed as analyses were restricted to abundant proteins, which would give the best chance for positive identification.

Metaproteomic approaches have thus far only been applied to laboratory scale bioreactors with a specialized community selected for phosphate removal [18] and a low-complexity natural microbial biofilm [21]. Extending this approach to complex environmental samples was not trivial. Initial studies comparing isolated strains, artificial communities and natural community samples by 1D-PAGE indicated that more resolving power was needed to deal with even simplified communities (data not shown). Thus, a metaproteomic approach utilizing 2D-PAGE and MS based protein identification was adopted. The experimental protocol outlined in this study was designed to avoid metaproteome changes arising from bias in the sample collection or handling. This was tested using artificial constructed bacterial assemblage containing 3 different species with varied cell sizes and we found no significant biases.

**Figure 6:**
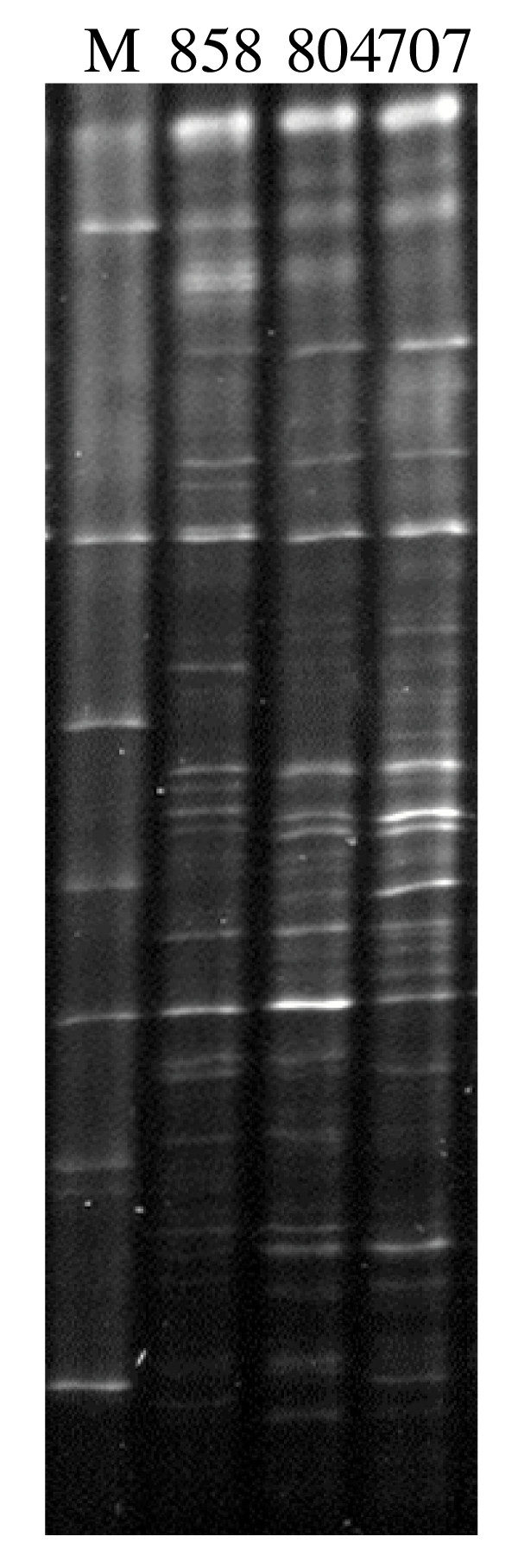
**DGGE fingerprints of bacterioplankton communities in Chesapeake Bay**. 858, 804 and 707 are sampling stations. M: marker.

The protocol was also field tested by comparing replicated samples from the middle Chesapeake Bay to each other and comparing a range of samples from upper, middle and lower Chesapeake Bay stations. The replicated samples shared more than ~92 % of proteins indicating that the metaproteomic approach applied in this study was robust. Furthermore, significant differences were noted when the middle Bay metaproteomes was compared with lower Bay and upper Bay metaproteomes with only 70 % and 30 % of protein spots in common. This pattern can be likely and partially explained by the difference among the population structures of these samples. Genetic fingerprints indicated that upper Bay bacterioplankton community was different from the middle and lower Bay (Fig. 6). Clustering analysis based on presence/absence of DGGE bands showed that the similarity between middle Bay to lower Bay was 64% while the upper Bay only shared 46% similarity to both of middle Bay and lower Bay. Finally, relative spot abundance was also much more tightly correlated when the replicated middle Bay samples were compared to each other than when they were compared to the lower Bay sample. These results demonstrate the approach outlined here is sufficiently sensitive to detect both coarse (shared spots) and fine (relative spot abundance) quantitative differences between samples, even when relatively low numbers of spots are included in the analysis. This is critical for any comparative approach.

This study, in addition to others, indicates that protein identification is the major challenge for metaproteomics [18,21,23,24]. Although distinct mass spectra from 34 protein spots were obtained by MALDI-TOF MS, no significant matches were found in sequence databases. MALDI-TOF generally requires at least 97 % amino acid sequence identity between query and target to find a significant match [[25], Hanson unpublished]. It seems unlikely that many proteins in environmental samples will share this level of identity with proteins in sequence databases derived from cultured organisms. Post-translational modifications of proteins also account for the difficulty in the identifications. Thus, MALDI-TOF MS is unlikely to be useful for metaproteomic approaches.

In contrast, LC-MS/MS or N-terminal sequencing coupled to MS-BLAST searching is able to provide tentative identification for metaproteomes. However, the abundance of most proteins is too low to be identified through the venue of N-terminal sequencing. In the community proteomic analysis of a natural acid mine drainage microbial biofilm, the proteins could be identified by MS and assigned to five most abundant microbes because of the availability of metagenomic data. But the relative high likelihood of false-positive protein identification requires matching of two or more peptides per protein for confident detection [21]. Therefore, caution is required for interpretation of the data. In this study, three Chesapeake Bay metaproteome samples matched different hypothetical proteins annotated in the Sargasso Sea metagenome [12]. This result strongly supports a marine origin for these sequences as would be expected for a large number of proteins in the Chesapeake Bay, particularly in lower and middle Bay samples where there is significant salinity. Even with tentative identities, extending that identity to function must be done with some care. The Sargasso Sea metagenome hypothetical protein corresponding to sample CB1 is not significantly similar to any known proteins in sequence databases giving no clues to its function. Sample CB3 may correspond to subunit 7 of the NADH:ubiquinone oxidoreductase or complex I (Table 3). Complex I is a key component of most membrane bound electron transport chains that is responsible for the transfer of electrons from cytoplasmic NADH pools to the membrane bound quinone pool coupled to proton motive force generation. Subunit 7 is a peripheral membrane protein of the quinone reduction core of complex I [26]. The organism containing the closest match is *Cytophaga hutchinsonii*, a member of the *Bacteroidetes *assemblage of organisms, which is a substantial fraction of many marine communities [27]. A current study on population structure of Chesapeake Bay bacterioplankton showed that *Bacteroidetes *group accounts for ~10% of total community in summer time [e. g. Kan unpublished].

Sample CB6 is similar to a family of predicted aminopeptidases with unspecified functional significance. The closest matching protein is from *Novosphingobium aromaticivorans*. While *N. aromaticivorans *is normally considered terrestrial, other *Novosphingobium *and related *Sphingobium *and *Sphingopyxis *strains are widely distributed. As an important component of the α-proteobacteria, these groups can be detected in and isolated from marine and estuarine environments [[28,29], Kan unpublished]. This identification along with that of CB3 support an aquatic bacterial origin for these proteins that is consistent with their presence in the Chesapeake Bay.

Unanswered questions remain regarding the applicability of metaproteomics to natural communities. These include the following: Does a focused protein spot on a 2D SDS-PAGE gel from an environmental sample contain one protein or multiple proteins? What type of information is required to infer identity of spots between different samples? What is the sensitivity of metaproteomics to changes in community composition and the physiological status of community members? How can functional inferences provided by metaproteomics be further tested? Will the approach outlined here be applicable to other systems such as soils, sediments, and extreme environments? Clearly, much more work and complementary approaches need to be applied to these problems.

## Conclusion

To our knowledge, this study represents the first application of a metaproteomic approach to a high-complexity aquatic microbial community. The main goals of this study were to develop a method capable of collecting planktonic microbial proteins in quantities suitable for analysis by 2D-PAGE. This was accomplished and attempts were made to identify a subset of these proteins. These attempts reinforced the notion that sequence based methods (LC-MS/MS) will be required to make any headway in protein identification in natural systems. Future studies will identify a much larger number of proteins from Chesapeake Bay microbial communities to address the questions raised above and provide insights into microbial community dynamics and function.

## Methods

### Bacterial cultures

Eight bacterial strains isolated from upper Chesapeake Bay (Baltimore Inner Harbor) were used in this study. Based on 16S rRNA gene sequences, these bacteria have been identified as *Vibrio vulnificus*, Marine *Bacillus *sp., *Marinomonas *sp., *Psychrobacter pacificens, Pseudomonas *sp., *Pseudoalteromonas *sp., *Shewanella *sp., and *Hahella *sp. respectively [Kan unpublished]. These bacteria were grown in 1/2 YTSS broth (4 g yeast extract, 2.5 g tryptone per liter dissolved in *in situ *water) and harvested at the exponential growth stage using centrifugation (10,000 × *g*, 5 min, 4°C).

### Artificial Community Construction and Recovery

To determine if microbial community analysis by 2D SDS-PAGE is feasible and representative, a simple artificial mixed community was constructed using three bacterial strains of differing size: *Chlorobium tepidum *strain WT2321 (~0.5–0.8 μm cell length), *Escherichia coli *strain JM109 (~1.2–1.6 μm cell length), and an uncharacterized strain of *Pseudomonas fluorescens *(~8–10 μm cell length) (kindly provided by G. A. O'Toole, Dartmouth University). Protein content per cell for each strain was determined by measuring protein via a modified Bradford assay (Bio-Rad) and direct cell counting on replicate samples for each organism. Communities containing the same amount of protein for each strain were constructed by mixing appropriate volumes of pure cultures. The mock community was then diluted into 5 1 of 10 mM potassium phosphate buffer (pH = 7.2) to specific cell densities and the cells recovered. Total protein extracts of the mock community and each member strain were made by pelleting cell samples in a microfuge and extracting proteins by resuspending in 5 M urea + 2 M thiourea + 2 % (w/v) CHAPS + 2 % (w/v) SB 3–10 + 40 mM Tris + 0.2 % (w/v) BioLyte 3–10 (sequential extraction reagent 3, Bio-Rad) at room temperature and vortexing for 2 minutes.

### Microbial community sampling

Picoplankton communities were collected at three stations along the middle axis of the Chesapeake Bay on 7 June 2003 aboard the R/V *Cape Henlopen *(Fig. 1). The stations 858, 804 and 707 represent the upper, middle and lower Bay, respectively. At each station, 0.2 g of chloramphenicol (Fisher Scientific, NJ) and 2 ml Protease inhibitor cocktail II (CalBiochem, CA) were added to 20 l of surface water (1 m below) to stop protein synthesis and inhibit activities of proteases. Samples were pre-filtered through 3-μm-pore-size polycarbonate filters (142-mm diameter; Millipore, Bedford, MA) to remove large particles and eukaryotes. The filter was replaced every 5 liters. Microbial cells in the filtrate were concentrated to a final volume of 150 ml using a tangential-flow ultrafiltration (30,000 MW cutoff) as described elsewhere [30]. Duplicate water samples were collected at station 804. Microbial cells in the retentate were pelleted using GS-15R centrifuge (Beckman, Fullerton, CA) at 13,000 × g, 4°C for 10 minutes. The collected cells were rinsed with TS washing buffer (Tris-HCl 10 mM, Sucrose 250 mM, pH 7.6) and resuspended with 0.5 ml of extraction buffer. The extraction buffer consisted of 0.01 M Tris-HCl, pH 7.4, 1 mM EDTA, 7 M urea and 2 M thiourea, 10% (v/v) glycerol, 2 % CHAPS, 0.2 % amphylotes, 0.002 M Tributyl phosphine (TBP), DNase (0.1 mg/ml), RNase (0.025 mg/ml) and proteinase inhibitor cocktail (CalBiochem, CA). TBP, DNase, RNase and proteinase inhibitor cocktail were freshly added to the buffer prior to applying to samples. Cells were stored frozen until further processing.

To estimate the recovery efficiency of ultrafiltration, bacterial cells were counted before and after ultrafiltration. Bacterial cells were stained with SYBR Gold (Molecular Probes, Inc., Eugene, Oreg.) following the protocol described previously [31]. Bacterial cells were enumerated under blue excitation (485 nm) on a Zeiss Axioplan epifluorescence microscope (Zeiss) using 63 × Antiflex Neoflua oil objective lens. At least 200 bacterial cells per sample were counted.

### Protein extraction and purification

For 1D-PAGE, proteins from natural microbial communities and cultured bacteria were extracted using lysis buffer (50 mM Tris-HCl, 2% SDS, 10% v/v glycerol, 0.1 M DTT, 0.01% Bromophenol Blue, pH 6.8). Cells suspended in buffer were heated in a boiling water bath for 2 minutes followed by centrifugation (10,000 × g, 4°C for 3 min). The supernatant was collected and 20 μg protein for each was loaded onto polyacrylamide gels. Silver staining was applied to 1D-PAGE gels.

For 2D-PAGE samples, cell suspensions were passed through a French Pressure cell (SLM Aminco) at 20,000 lb/in^2 ^twice and then incubated on ice for 20 minutes. During the ice incubation, samples were vortexed for 15 sec every 5 minutes. Large cellular debris was removed by centrifugation (10,000 × *g*, 4°C for 5 min). Proteins in the supernatant were precipitated with trichloracetic acid and resuspended in extraction buffer. Protein concentration of the sample was determined using the RC DC protein assay kit (Bio-Rad, Hercules, CA). Extracted proteins were stored at -80°C.

### Isoelectric Focusing (IEF) and SDS-PAGE

The first dimension separation of proteins was carried out in the immobilized pH gradient (IPG) strips (11 cm, pH 3–10 or 4–7) on a Bio-Rad Protean IEF Cell system (Bio-Rad, Hercules, CA). Each 2D-PAGE was conducted using 100 μg of total protein. The IEF program was: 250V for 20 min followed with a linear ramp to 8000V for 2.5 hr, and 8000V for a total 40,000 V-hr with a rapid ramp. After the first dimension, the IEF strips were equilibrated in freshly made Buffer 1 (6 M urea, 2% SDS, 0.05 M Tris/HCl pH 8.8, 50% Glycerol) and Buffer 2 (6 M urea, 2% SDS, 0.375 M Tris/HCl pH 8.8, 20% Glycerol and 0.5 g iodoacetamide) (Bio-Rad, Hercules, Calif), respectively.

The second dimension of 2D-PAGE were performed using 8–16% gradient precast polyacrylamide gels (Bio-Rad, Hercules, CA) following the manufacturer's instructions. The gels were stained with SYPRO Ruby (Bio-Rad, Hercules, CA) after electrophoresis and scanned using a Typhoon 9410 fluorescent Imager (Amersham, NJ) with 488nm excitation and emission filter 610 BP30.

### Metaproteome Image Analysis

Images were analyzed and quantitatively compared using the Z3 proteomics software package (Compugen, Israel). Gel images were compared in multiple gel mode using the total density in gel method for spot quantification. All gels were subjected to the same spot detection parameters followed by automated matching. Pairwise comparisons of gels were inspected and matches edited manually to eliminate poor quality or low intensity matches. When automatic matching failed, the number of matched and unmatched spots was estimated by manual examination of overlaid 2D SDS-PAGE images.

### Protein Identification by Mass Spectrometry

Protein spots were manually excised from gels using Pasteur pipettes and digested as described by Mann et al. [32]. Tryptic peptides were analyzed both via MALDI-TOF and LC-MS/MS. MALDI spectra were acquired on a Bruker (Billerica, MA) Biflex III MALDI mass spectrometer operating in reflectron mode with delayed extraction. External calibration was performed using Calibration Mixture 2 from the Sequazyme Peptide Mass Standards Kit (Applied Biosystems, Foster City, CA). LC-MS/MS was performed on a Micromass (Beverly, MA) Q-TOF Ultima API-US coupled to a Micromass capLC. Tryptic digests were separated using both a C18 trapping column for washing and concentrating (LC Packings (Sunnyvale, CA) 300 μm × 5 mm C18) and a C18 analytical column for enhanced separation (LC Packings 180 μm × 15 cm C18). The solvent system consisted of 95% 0.1% formic Acid, 5% acetonitrile for the aqueous phase and 95% acetonitrile, 5% 0.1% formic Acid for the organic phase. A 60/60 gradient (to 60% organic in 60 mins) running at l μl/min was employed with most peptides eluting by ~30% organic. The LC eluent was electrosprayed directly into the Q-TOF using the nanosprayer source. Data dependent scanning was used with both MS and MS/MS spectra being acquired during an LC run. Spectra were processed and deconvoluted using programs found with the Micromass operating system, MassLynx v. 3.5.

MALDI-TOF peak lists were searched against protein sequence databases using the Matrix Science Mascot web interface http://www.matrixscience.com/search_form_select.html. Deconvoluted MS/MS spectra were analyzed using a demonstration version of PeaksStudio 3.0 software (Bioinformatics Solutions Inc., Canada) for *de novo *sequence prediction. All sequences for each protein spot were used as queries in MS-BLAST searches as described by Shevchenko et al. [33] via the MS-BLAST web interface http://dove.embl-heidelberg.de/Blast2/msblast.html.

## Competing interests

The author(s) declare that they have no competing interests.

## References

[B1] Campbell L Nolla HA Vaulot D The importance of *Prochlorococcus *to community structure in the central North Pacific Ocean Limnol Oceanogr 1994 39 954 961

[B2] Li WKW Primary productivity of prochlorophytes, cyanobacteria, and eucaryotic ultraphytoplankton: measurements from flow cytometric sorting Limnol Oceanogr 1994 39 169 175

[B3] Hobbie JE Daley RJ Jasper J Use of Nuclepore filters for counting bacteria by fluorescence microscopy Appl Environ Microbiol 1977 33 1225 1228 327932 10.1128/aem.33.5.1225-1228.1977 PMC170856

[B4] Azam F Microbial control of oceanic carbon flux: The plot thickens Science 1998 280 694 696 10.1126/science.280.5364.694

[B5] Giovannoni SJ Britschgi TB Moyer CL Field KG Genetic diversity in Sargasso Sea bacterioplankton Nature 1990 345 60 63 10.1038/345060a0 2330053

[B6] Amann RI Ludwig W Schleifer K Phylogenetic identification and in situ detection of individual microbial cells without cultivation Microbiol Rev 1995 59 143 169 7535888 10.1128/mr.59.1.143-169.1995 PMC239358

[B7] Rappe MS Cannon SA Vergin KL Giovannoni SJ Cultivation of the ubiquitous SAR11 marine bacterioplankton clade Nature 2002 418 630 633 10.1038/nature00917 12167859

[B8] Ward DM Weller R Bateson MM 16S rRNA sequences reveal numerous uncultured microorganisms in a natural community Nature 1990 345 63 65 10.1038/345063a0 1691827

[B9] Beja O Suzuki MT Koonin EV Aravind L Hadd A Nguyen LP Villacorta R Amjadi M Garrigues C Jovanovich SB Feldman RA DeLong EF Construction and analysis of bacterial artificial chromosome libraries froma marine microbial assemblage Environ Microbiol 2000 2 516 529 10.1046/j.1462-2920.2000.00133.x 11233160

[B10] Rondon MR August PR Bettermann AD Brady SF Grossman TH Liles MR Loiacono KA Lynch BA MacNeil IA Minor C Tiong CL Gulman M Osburne MS Clardy J Handelsman J Goodman RM Cloning the soil metagenome: a strategy for accessing the genetic and functional diversity of uncultured microorganisms Appl Environ Microbiol 2000 66 2541 2547 10.1128/AEM.66.6.2541-2547.2000 10831436 PMC110579

[B11] Tyson GW Chapman J Hugenholtz P Allen EE Ram RJ Richardson PM Solovyev VV Rubin EM Rokhsar DS Banfield JF Community structure and metabolism through reconstruction of microbial genomes from the environment Nature 2004 428 37 43 10.1038/nature02340 14961025

[B12] Venter JC Remington K Heidelberg JF Halpern AL Rusch D Eisen JA Wu D Paulsen I Nelson KE Nelson W Fouts DE Levy S Knap AH Lomas MW Nealson K White O Peterson J Hoffman J Parsons R Baden-Tillson H Pfannkoch C Rogers Y Smith HO Environmental genome shotgun sequencing of the Sargasso Sea Science 2004 304 66 74 10.1126/science.1093857 15001713

[B13] Lopez MF Proteome analysis I. Gene products are where the biological action is J Chromatogr 1999 722 191 202 10068141

[B14] Petersohn A Brigulla M Haas S Hoheisel JD Volker U Hecker M Global analysis of the general stress response of *Bacillus subtilis* J Bacteriol 2001 183 5617 5631 10.1128/JB.183.19.5617-5631.2001 11544224 PMC95453

[B15] Eymann C Homuth G Scharf C Hecker M *Bacillus subtilis *functional genomics: global characterization of the stringent response by proteome and transcriptome analysis J Bacteriol 2002 184 2500 2520 10.1128/JB.184.9.2500-2520.2002 11948165 PMC134987

[B16] Conway T Schoolnik GK Microarray expression profiling: capturing a genome-wide protrait of the transcriptome Mol Microbiol 2003 47 879 889 10.1046/j.1365-2958.2003.03338.x 12581346

[B17] Blackstock WP Weir MP Proteomics: quantitative and physical mapping of cellular proteins Trends Biotechnol 1999 17 121 127 10.1016/S0167-7799(98)01245-1 10189717

[B18] Wilmes P Bond PL The application of two-dimensional polyacrylamide gel electrophoresis and downstream analyses to a mixed community of prokaryotic microorganisms Enivron Microbiol 2004 6 911 920 10.1111/j.1462-2920.2004.00687.x 15305916

[B19] Ogunseitan OA Direct extraction of catalytic proteins from natural microbial communities J Microbiol Methods 1997 28 55 63 10.1016/S0167-7012(96)00962-1

[B20] Ogunseitan OA Extraction of proteins from aquatic environments Mol Microbiol Ecol Mann 1998 4.1.6 1 12

[B21] Ram RJ VerBerkmoes NC Thelen MP Tyson GW Baker BJ Blake RC II Shah M Hettich RL Banfield JF Community proteomics of a natural microbial biofilm Science 2005 308 1915 1920 10.1126/science.1109070 15879173

[B22] Zubkov MV Fuchs BM Eilers H Burkill PH Amann R Determination of total protein content of bacterial cells by SYPRO staining and flow cytometry Appl Environ Microbiol 1999 65 3251 3257 10388732 10.1128/aem.65.7.3251-3257.1999 PMC91485

[B23] Liska AJ Shevchenko AJ Combining mass spectrometry with database interrogation strategies in proteomics Trends Anal Chem 2003 22 291 299 10.1016/S0165-9936(03)00507-7

[B24] Habermann B Oegeman J Sunyaev S Shevchenko AJ The power and the limitations of cross-species protein identification by mass spectrometry-driven sequence similarity searches Mol Cell Proteomics 2004 3 238 249 10.1074/mcp.M300073-MCP200 14695901

[B25] Shevchenko A Sunyaev S Liska A Bork P Nanoelectrospray tandem mass spectrometry and sequence similarity searching for identification of proteins from organisms with unknown genomes Methods Mol Biol 2003 211 221 234 12489434 10.1385/1-59259-342-9:221

[B26] Zickermann V Bostina M Hunte C Ruiz T Radermacher M Brandt U Functional implications from an unexpected position of the 49-kDa subunit of NADH:ubiquinone oxidoreductase J Biol Chem 2003 278 29072 29078 10.1074/jbc.M302713200 12754256

[B27] Cottrell MT Kirchman DL Community composition of marine bacterioplankton determined by 16S rRNA gene clone libraries and fluorescence in situ hybridization Appl Environ Microbiol 2000 66 5116 5122 10.1128/AEM.66.12.5116-5122.2000 11097877 PMC92431

[B28] Ostrowski M Fegatella F Wasinger V Guilhaus M Corthals GL Cavicchioli R Cross-species identification of proteins from proteome profiles of the marine oligotrophic ultramicrobacterium, *Sphigopyxis alaskensis* Proteomics 2004 4 1779 1788 10.1002/pmic.200300695 15174144

[B29] Sohn JH Kwon KK Kang JH Jung HB Kim SJ *Novosphingobium pentaromativoram *sp. nov., a high-molecular-mass polycyclic aromatic hydrocarbon-degrading bacterium isolated from estuarine sediments Int J Syst Evol Microbiol 2004 54 1483 1487 10.1099/ijs.0.02945-0 15388699

[B30] Chen F Suttle CA Short SM Genetic diversity in marine algal virus communities as revealed by sequence analysis of DNA polymerase genes Appl Environ Microbiol 1996 62 2869 2874 8702280 10.1128/aem.62.8.2869-2874.1996 PMC168073

[B31] Chen F Lu J Binder BJ Liu Y Hodson RE Application of digital image analysis and flow cytometry to enumerate marine viruses stained with SYBR Gold Appl Environ Microbiol 2001 67 539 545 10.1128/AEM.67.2.539-545.2001 11157214 PMC92618

[B32] Shevchenko A Wilm M Vorm O Mann M Mass spectrometric sequencing of proteins from silver-stained polyacrylamide gels Anal Chem 1996 68 850 858 10.1021/ac950914h 8779443

[B33] Shevchenko A Sunyaev S Loboda A Bork P Ens W Standing KG Charting the proteomes of organisms with unsequenced genomes by MALDI-quadrupole time-of-flight mass spectrometry and BLAST homology searching Anal Chem 2001 73 1917 1926 10.1021/ac0013709 11354471

